# The Vaginal and Urinary Microbiomes in Premenopausal Women With Interstitial Cystitis/Bladder Pain Syndrome as Compared to Unaffected Controls: A Pilot Cross-Sectional Study

**DOI:** 10.3389/fcimb.2019.00092

**Published:** 2019-04-08

**Authors:** Kate V. Meriwether, Zhenmin Lei, Rajbir Singh, Jeremy Gaskins, Deslyn T. G. Hobson, Venkatakrishna Jala

**Affiliations:** ^1^Department of Obstetrics and Gynecology, University of Louisville, Louisville, KY, United States; ^2^Department of Microbiology and Immunology, James Graham Brown Cancer Center, University of Louisville, Louisville, KY, United States; ^3^Department of Bioinformatics and Biostatistics, University of Louisville, Louisville, KY, United States

**Keywords:** interstitial cystitis, painful bladder, bladder pain syndrome, microbiome, urine, vagina, genitourinary

## Abstract

Interstitial cystitis/bladder pain syndrome (ICBPS) may be related to an altered genitourinary microbiome. Our aim was to compare the vaginal and urinary microbiomes between premenopausal women with ICBPS and unaffected controls. This cross-sectional study screened premenopausal women with an O'Leary-Sant questionnaire (ICBPS if score ≥6 on either index; controls <6 on both). Women completed questionnaires on health characteristics, pelvic floor symptoms (OABq, PFDI-20), body image (mBIS), and sexual function (PISQ-IR). Bacterial genomic DNA was isolated from vaginal and clean-catch urinary specimens; the bacterial 16 rRNA gene was sequenced and analyzed using the QIIME pipeline. We performed UniFrac analysis (β-diversity) and generated Chao1 estimator (richness) and Simpson index (richness and evenness) values. We analyzed 23 ICBPS and 18 non-ICBPS patients. ICBPS patients had increased vaginal deliveries, BMI, and public insurance as well as worsened OAB-q, PFDI-20, mBIS, and PISQ-IR domain scores. *Lactobacilli* was the most abundant genus in both cohorts, and anaerobic or fastidious predominance was similar between groups (*p* = 0.99). For both the urine and vagina specimens, Chao1 and Simpson indices were similar between ICBPS and unaffected women. Weighted and unweighted UniFrac analyses showed no differences between groups. A significant correlation existed between the urinary and vaginal Simpson indices in ICBPS women, but not in unaffected women. Premenopausal women with ICBPS, despite worsened socioeconomic indicators and pelvic floor function, were not found to have significantly different urinary and vaginal microbiomes compared to women without ICBPS.

## Introduction

Interstitial cystitis/bladder pain syndrome (ICBPS) is a devastating and common disorder in women (Clemens et al., 2005). The pathophysiology of ICBPS is poorly understood (Patnaik et al., 2017), leading to an ineffectual treatment environment (Kanter et al., 2017). There is a need to find underlying etiologies and direct that knowledge at therapy (Hanno et al., 2011).

Microbial studies indicate that women with ICBPS may have urinary microbiome disturbances (Siddiqui et al., 2012; Nickel et al., 2016; Abernethy et al., 2017), and it is known that disturbances in the vaginal microbiome can alter the urinary microbes (Gottschick et al., 2017; Thomas-White et al., 2018). As ICBPS onset is often in the reproductive years (Clemens et al., 2012), microbial interactions with ICBPS in pre-menopausal women is an important focus of study. However, no published research integrates both urinary and vaginal niches into the microbial study of ICBPS pathology.

Our primary aim was to compare the urinary and vaginal microbiomes between two groups: pre-menopausal women with ICBPS and those women unaffected by ICBPS. Based on past study that women with urinary symptoms more altered species compared to healthy women (Pearce et al., 2014; Karstens et al., 2016), we hypothesized that women with ICBPS would have a more anaerobic and fastidious bacterial milieu than unaffected controls.

## Materials and Methods

Premenopausal women were recruited from a subspecialty practice at the University of Louisville and underwent screening assessment with the O'Leary-Sant questionnaire (Lubeck et al., 2001). Women were eligible if they were hormonally pre-menopausal and excluded if they had issues that disturbed the microbiome of the genitourinary system, were hormonally post-menopausal, or unable to understand the consent process (Appendix 1).

Women who scored <6 points on both indices of the O'Leary-Sant questionnaire were considered unaffected, non-ICBPS participants, and women with a score ≥6 on either index were considered ICBPS participants. The study was approved by the Institutional Review Board, and women underwent an informed consent prior to participation. All participants had a midstream, clean-catch urinary specimen sent for microscopic urinalysis (mUA) with reflex urine culture if indicated. The urinary specimen was collected after a front to back wipe with a moistened gynecological cleaning towelette, with the patient instructed to void for 1–2 s prior to collection in the sterile cup. If the women were found to have urinary infection or other exclusion criteria after the study visit (Appendix 1), they were excluded.

Participating patients completed questionnaires on their characteristics, health history, use of tobacco, vaginal medications, hormones/contraceptives, or probiotics. If possible, we calculated the days since their last menstrual cycle (LMP). Patients completed the pelvic floor distress inventory (PFDI-20), overactive bladder questionnaire (OAB-q), modified body image scale (mBIS), and pelvic organ prolapse/incontinence sexual questionnaire: IUGA revised (PISQ-IR). Women underwent collection of a BD ProbeTec^TM^ swab (Becton Dickinson, Franklin Lakes, NJ) from the mid-vaginal walls and a clean-catch, midstream urinary specimen in a sterile cup (separate from mUA specimen). Vaginal and urinary samples were both sealed and stored in refrigeration (4°C) for <12 h prior to laboratory processing.

At the laboratory, urine specimens were sampled after agitation by pipetting three aliquots from each specimen (1.0 mL each) into three 1.5 mL nucleic acid-free tubes. Tubes were centrifuged at 12,000 g for 10 min and the supernatant discarded (Pearce et al., 2014; Abernethy et al., 2017). The pellet was suspended in a PBS buffer, centrifuged again for 5 min at 12,000 g, and the remaining supernatant was removed.

At the laboratory, vaginal samples were also placed in −80°C until pellet extraction, where thawed swabs were agitated for 30 s in a tube containing 1 mL of sterile PBS and pressed against wall of the tube multiple times. The pellet was isolated by centrifuging the solution at 12,000 g for 5 min, and the supernatant was discarded.

Urine and vaginal pellets were stored at −80°C until DNA extraction. A bead based extraction method was used for isolation of the total bacterial genomic DNA (gDNA) from the pellets using a QIAmp BiOstic Bacteremia DNA kit (Qiagen Inc, Germantown, MD).

The hypervariable V3-V4 regions of the 16S rRNA gene were amplified using preexisting primers (Integrated DNA Technologies, Skokie, Illinois) as follows:

Forward Primer: 5′TCGTCGGCAGCGTCAGATGTGTATAAGAGACAGCCTACGGGNGGCWGCAG3′

Reverse Primer: 5′GTCTCGTGGGCTCGGAGATGTGTATAAGAGACAGGACTACHVGGGTATCTAATCC3′

We utilized 12.5 ngmicrobial genomic DNA with appropriate PCR conditions, and an extraction-negative controlwas run with amplification and sequencing steps for vaginal and urinary specimens to ensure lack of laboratory contamination. Each PCR reaction was performed in triplicate and then pooled with respective samples. The amplified bacterial gDNA wasquantified using NanoDrop8000(ThermoScientific, Waltham, MA). ThePCR products were cleaned using AMPure XP beads. These PCR products weresubjected to index PCR to attach dual indices and Illumina (San Diego, CA) sequencing adapters usingthe Nextera XT kit (FC-121-1012). The resulting products were again cleaned using AMPure XP beads and quantified by usingQubit dsDNA BR Assay kit (ThermoScientific, Waltham, MA). The samples (4 nM) were pooled and denatured to perform sequencing usingMiSeq protocols alongside a control library. The sequencing was performed usingthe Illumina MiSeq Reagents kit v3(600 cycles; MS-102-3003) on the MiSeqinstrument (Illumina, San Diego, CA).

Microbiome data were pre-processed with Microbiome Helper v1.0.2 (Comeau et al., 2017). Reads were stitched and filtered for quality reads. Paired-end reads were stitched using PEAR v0.0.10 (Zhang et al., 2014). Reads with >10% of bases having a quality score of ≤30 were removed using FASTX-Toolkit. Reads ≥400 base-pairs that contained the matching primer sequences were retained. Following this, chimeric sequences were removed using the implementation of the UCHIME algorithm (Edgar et al., 2011) in VSEARCH v2.4.4 (Rognes et al., 2016).

Sequences were grouped into Operational Taxonomic Units (OTU's) using QIIME v1.9.1 (Navas-Molina et al., 2013). We grouped reads into OTUs at 97% similarity using an open-reference approach in QIIME. Reads were first clustered with the algorithm SortMeRNA (Kopylova et al., 2012) against the Greengenes database (v13_8) (DeSantis et al., 2006). This was followed by *de novo* clustering with SUMACLUST (v1.0.00) (Mercier and Coissac, 2013). The OTUs were normalized by random subsampling (rarefying) to 5,000 and 10,000 reads per sample. We analyzed the data that was rarefied to a depth of 5,000 sequences/sample, as 10,000 sequences/sample was not found more informative and eliminated viable data (Figure 2).

The primary outcome was the presence of an anaerobic or fastidious organisms as the predominant genera of the sample (>50% of reads), based on past study indicating an increase in these microorganisms to the urinary microbiome of women with urinary disease (Pearce et al., 2014; Karstens et al., 2016). We defined anaerobic or fastidious bacteria as those including *Prevotella, Mobiculus, Gardnerella vaginalis, Mycoplasma, Ureaplasma, Bacteroides*, and aerobic bacteria that are not classically cultivated in culture^1^. This was a pilot study without prior data on the topic for power calculation; we aimed to recruit 20 patients per group for a convenience sample.

We calculated two measures of alpha diversity for each sample (urine and vagina of each patient) using QIIME: the Chao1 estimator (richness) and the Simpson index (evenness and richness). The McIntosh index of evenness and the Shannon index (evenness and richness) were also calculated for each sample as additional measures of alpha diversity. Beta diversity principle co-ordinate plots (PCOs) were generated using phylogenetic UniFrac distances (Lozupone and Knight, 2005). Permanova analyses (weighted and unweighted) calculated the significance of Unifrac distances between the two groups. The percentile of reads of a certain genus for the sample (out of the total reads in the sample) was calculated for the top 5 most predominant genera, as beyond the top 5 genera the percentiles were so minute (<1%) that comparisons between them would not provide meaningful data.

To compare between ICBPS and control groups, we utilized chi-square (Fisher's test for n<5) for categorical variables and *t*-tests for continuous, parametric variables (Mann-Whitney for non-parametric). The multivariate Hotelling's *t*-test was used to compare measures of alpha diversity (Chao1 estimator and Simpson index of both urine and vaginal samples) between the groups. If data for the Chao1 estimator and Simpson index were skewed (violated parametric assumptions), these were analyzed on the logarithmic scale. The ANOSIM (analysis of similarity) test was utilized on the data as well without logarithmic transformation to account for the non-parametric nature of these outcomes.

Kendall correlation measures were used to analyze interactions between different sample locations (urine and vagina) for alpha diversity measures within the separate groups (ICBPS and unaffected). The relationships between baseline patient characteristics or questionnaire scores (OABq, PFDI, mBIS, and PISQ-IR) and microbial diversity were analyzed utilizing marginal univariate regression models, considering the pairs of urinary and vaginal alpha measures separately due to low power. To correct for multiple comparisons, we determined significance by applying an FDR correction across all regressions with the same measure (Chao or Simpson) and sample site (urinary or vagina). For covariates affecting the alpha measures at the 0.20 FDR level, we prepared to create a multiple regression model to explore the effect of group (ICBPS or unaffected) controlling for discovered confounders. All statistical analyses were performed using the R statistical software, version 3.3.1.

Stacked bar plots for the top 10 genus classifications for the urinary and vaginal samples of ICBPS women and non-ICBPS women were created using cluster 3.0 following Euclidean distance hierarchical matrices. Images were generated with Java Tree Software^2^.

## Results

**Figure 1:**
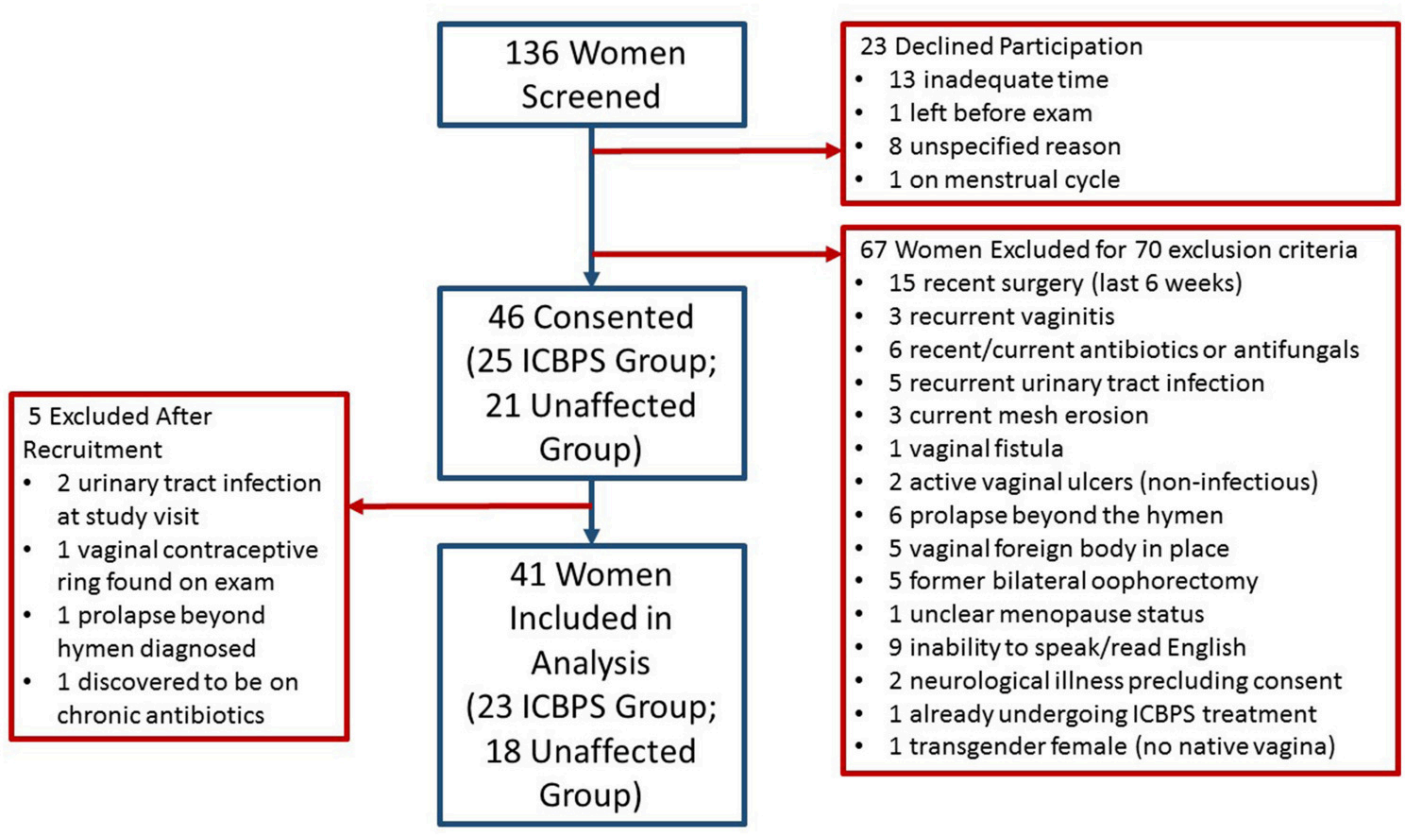
Summary of screening and inclusion of study population.

We screened 136 women for participation and 46 patients enrolled (Figure 1). Of women who enrolled, their presenting pelvic floor complaints included urinary incontinence (*n* = 12 ICBPS; *n* = 5 control), pelvic pain (*n* = 8 ICBPS; *n* = 4 control), vaginal prolapse (*n* = 5 ICBPS; *n* = 2 control), recurrent urinary infections (*n* = 2 ICBPS; *n* = 0 control), and other pelvic complaints (*n* = 0 ICBPS; *n* = 4 control)., Four women without pelvic floor complaints volunteered from the community and were enrolled in the control cohort. Five women were excluded after enrollment due to urinary tract infections (*n* = 2) or other exclusion criteria discovered after the study visit (*n* = 3), leaving 41 women (23 ICBPS; 18 unaffected) in the analyses. One woman in the non-ICBPS group had an uninterpretable vaginal swab and urine microbiome measures that were extreme outliers, so her samples were excluded from alpha and beta diversity analyses.

The mean days since the LMP was 22.2 days in the ICBPS group and 18.3 days in the unaffected group. However, this was based on only 12 women in each group, due lack of data on LMP (*n* = 1 ICBPS; *n* = 4 control) or with past procedures (hysterectomy, endometrial ablation, or hormonal implant) that precluded menstruation (*n* = 10 ICBPS; *n* = 2 control) in other patients. This variable of mean days since LMP did not have a significant impact on any microbiome outcomes (*p*-values 0.39–0.72).

The ICBPS patients and non-ICBPS patients had similar age, racial distribution, smoking status, sexual activity, hormone use, history of pelvic surgery/treatment, and Charleston health index scores (Table 1). However, ICBPS patients had increased vaginal deliveries, BMI, and public insurance as well as worsened scores on the OAB-q, PFDI-20, mBIS, and several PISQ-IR domains in sexually active women.

**Table 1:** Patient characteristics in the study groups and relationship of characteristics and outcomes to the vaginal and urinary microbiome.

**Characteristic**	**Entire study group *n*(%), mean ± SD, or median[IQR] (*n* = 41)**	**ICBPS group *n*(%), mean ± SD, or median[IQR] (*n* = 23)**	**Unaffected, non-ICBPS group *n*(%), mean ± SD, or median[IQR] (*n* = 18)**	***p*-value, comparison between groups**	*p*-value, relationship to urinary Chao1 estimator^*^	*p*-value, relationship to urinary Simpson index^*^	*p*-value, relationship to vaginal Chao1 estimator^*^	*p*-value, relationship to vaginal Simpson index^*^
Age	33.61 ± 8.97	33.56 ±7.87	33.67 ± 10.44	0.97	0.32	0.58	0.73	0.37
Parity	1 [0–3]	2 [1–3]	1 [0–2]	0.03	0.93	1.00	0.99	0.92
Vaginal deliveries	1 [0–2]	1 [0–3]	0 [0–1]	0.02	0.92	0.74	0.98	0.27
BMI (kg/m^2^)	30.90 ± 8.58	34.43 ± 8.34	26.38 ± 6.67	<0.01	0.42	0.22	0.38	0.23
Race/ethnicity				0.12	0.34	0.51	0.70	0.71
Caucasian	31 (76)	20 (87)	11 (61)					
African American	6 (15)	3 (13)	3 (17)					
Hispanic	1 (2)	0	1 (6)					
Asian	2 (5)	0	2 (11)					
Other	1 (2)	0	1 (6)					
Smoking	5 (12)	3 (13)	2 (11)	0.99	0.28	0.11	0.60	0.20
Probiotic use	6 (15)	3 (13)	3 (17)	0.99	0.37	0.23	0.40	0.37
Vaginal product use (e.g., douches)	8 (20)	7 (30)	1 (6)	0.06	0.64	0.77	0.28	0.53
O'Leary-Sant Total Score	13.80 ± 10.39	22.04 ± 5.43	3.28 ± 2.56	<0.01	0.98	0.30	0.87	0.41
OABq Symptom Score	16.93 ± 8.51	22.74 ± 6.48	9.5 ± 3.47	<0.01	0.68	0.19	0.88	0.89
OABq HRQOL	31.59 ± 16.74	42.88 ± 12.73	17.17 ± 7.55	<0.01	0.40	0.20	0.25	0.46
POPDI-6	21.95 ± 20.48	34.42 ± 17.82	6.02 ± 9.82	<0.01	0.45	0.94	0.35	0.28
CRADI-8	12.41 ± 16.87	19.27 ± 18.8	3.65 ± 8.18	<0.01	0.29	0.55	0.04	0.24
UDI-6	40.57 ± 29.86	58.99 ± 22.61	17.04 ± 19.79	<0.01	0.33	0.32	0.37	0.86
PFDI Total Score	74.93 ± 58.39	112.68 ± 48.15	26.7 ± 25.14	<0.01	0.28	0.48	0.17	0.54
mBIS	10.07 ± 7.08	13.83 ± 6.24	5.28 ± 4.92	<0.01	0.83	0.06	0.70	0.08
**PISQ- IR DOMAINS**								
Arousal	13.14 ± 3.15	12.11 ± 2.47	14.29 ± 3.48	0.04	0.71	0.41	0.25	0.22
Partner related	11.03 ± 1.87	10.53 ± 2.37	11.59 ± 0.87	0.08	0.92	0.38	0.76	0.31
Condition specific	13.17 ± 1.98	12.11 ± 1.92	13.76 ± 1.92	0.09	0.81	0.91	0.95	0.82
Global quality rating	12.42 ± 4.87	10.63 ± 4.39	14.41 ± 4.72	0.02	0.89	0.74	0.50	0.64
Condition impact	12.36 ± 3.57	10.68 ± 3.43	14.24 ± 2.75	<0.01	0.58	0.07	0.44	0.15
Desire	8.69 ± 2.78	8.32 ± 2.67	9.12 ± 2.91	0.40	0.67	0.93	0.89	0.94

* *p-values from the marginal univariate regression for the relationship between the variable (left column) and the two measures of alpha diversity (Chao1 and Simpson)*.

*IQR, inter-quartile range; ICBPS, interstitial cystitis/bladder pain syndrome; BMI, body mass index; OABq, overactive bladder questionnaire; HRQOL, health-related quality of life, POPDI, pelvic organ prolapse distress inventory; CRADI, colorectal-anal distress inventory; UDI, urogenital distress inventory; PFDI, pelvic floor distress inventory; PISQ-IR, pelvic organ prolapse and incontinence sexual function questionnaire, IUGA revision; mBIS, modified body image scale*.

We obtained 5,755,661 total quality sequence reads from 81 samples (average 71,058 reads/sample; 53,387 for urinary specimens and 89,170 for vaginal specimens). There were significantly more reads/sample in the vaginal space as opposed to the urinary space in this population (p<0.01). The average number of reads/sample for ICBPS women was 75,794, and for normal women was 64,832, which was not different between the groups (*p* = 0.31). The average reads/sample for urinary samples was not different between groups (ICBPS 56,070 and non-ICBPS 49,959, *p* = 0.62), and the average reads/sample for vaginal samples was also similar between groups (ICBPS 95,18 and non-ICBPS 80,580, *p* = 0.36).

**Figure 2:**
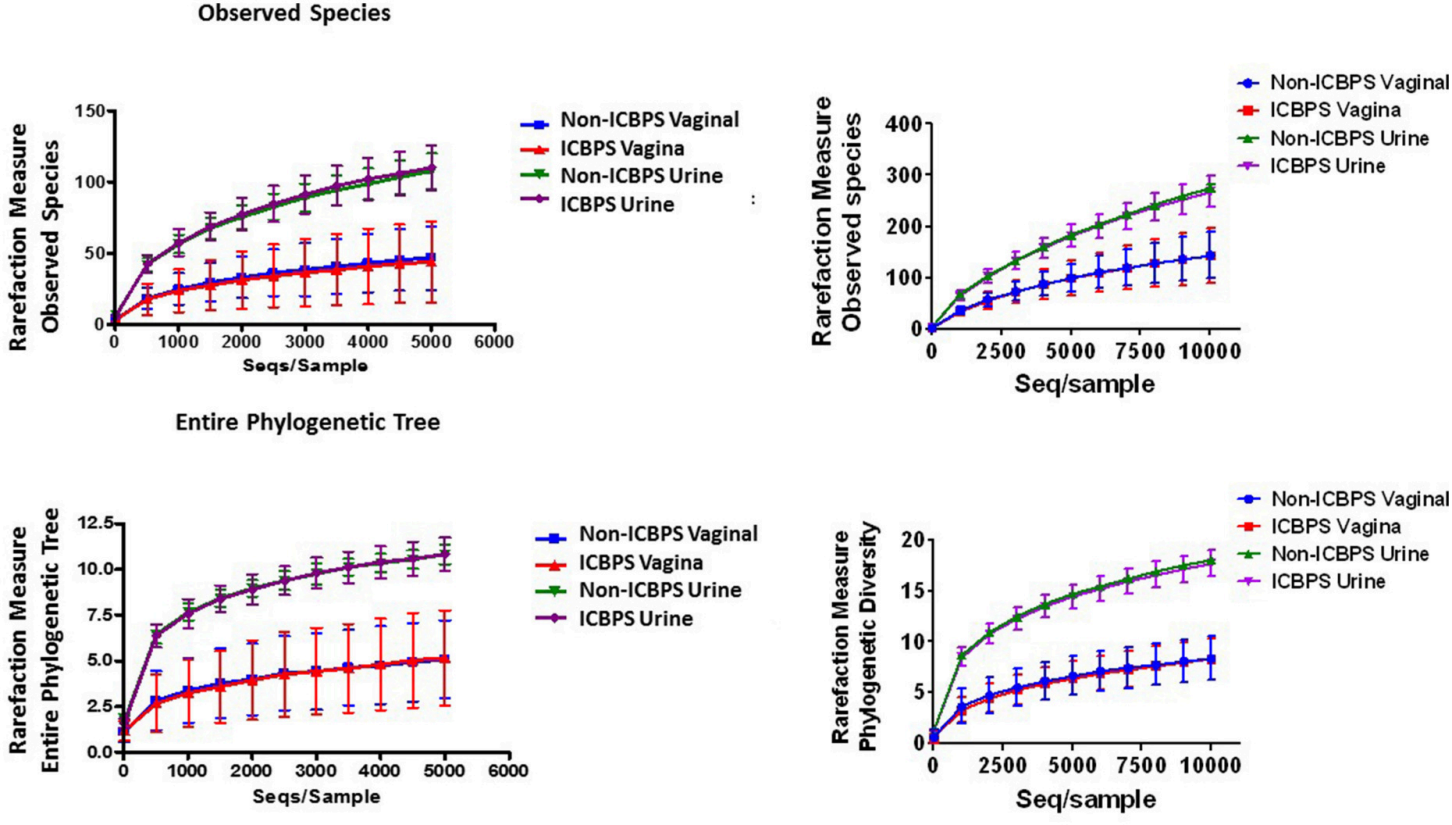
Plotting of rarefaction measure (y-axis) against sequences per sample (x-axis), either for observed species (top graphs) or the entire phylogenetic tree (bottom graphs), for each sample location (urinary or vaginal) and each group (ICBPS or non-ICBPS women) up to 5,000 sequences per sample **(left graphs)** or up to 10,000 sequences per sample **(right graphs)**.

**Figure 3:**
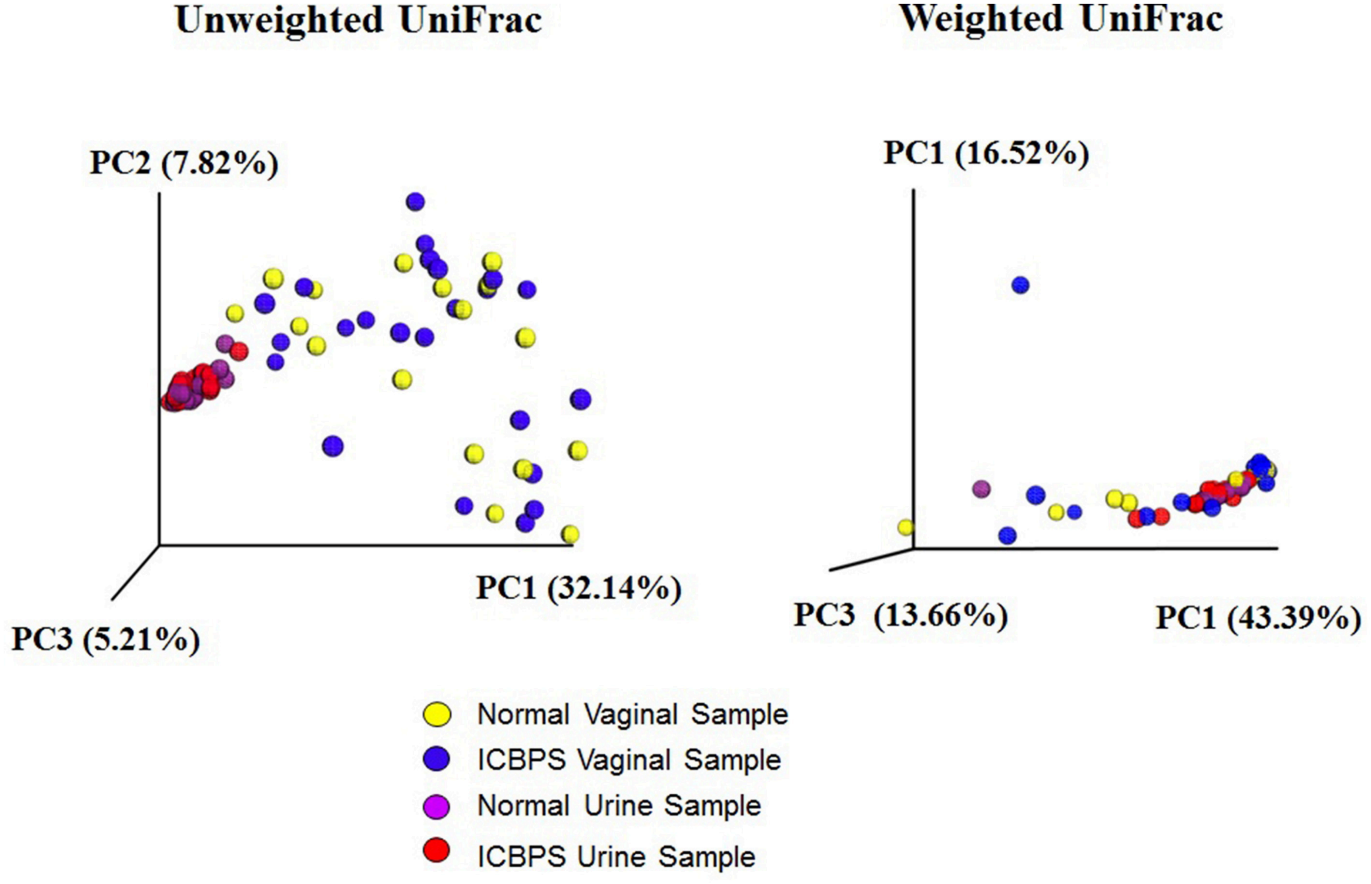
Unweighted and weighted UniFrac analyses with 3D plotting by primary component (PC) distances of normal vaginal (yellow) and normal urine (purple) samples as well as ICBPS women vaginal (blue) and urine samples (red).

One vaginal and 3 urine pellet samples contained <10,000 reads, and increasing rarefaction to 10,000 eliminated these data without substantially changing results (Figure 2), so rarefaction at 5,000 reads/sample was maintained. Urinary and vaginal microbiomes for observed species was similar between ICBPS women to non-ICBPS women, but urinary and vaginal samples were distinct from one another in both groups with rarefaction up to 5,000 reads/sample, and this did not change at up to 10,000 reads per sample (Figure 2). We did not detect significant differences between ICBPS and non-ICBPS women in beta diversity (multivariate Hotelling test, *p* = 0.58; ANOSIM *p* = 0.90). UniFrac analyses, both weighted and unweighted, yielded no significance in beta diversity between the ICBPS and control groups for the urine or the vagina (all *p* > 0.05, Figure 3).

**Figure 4:**
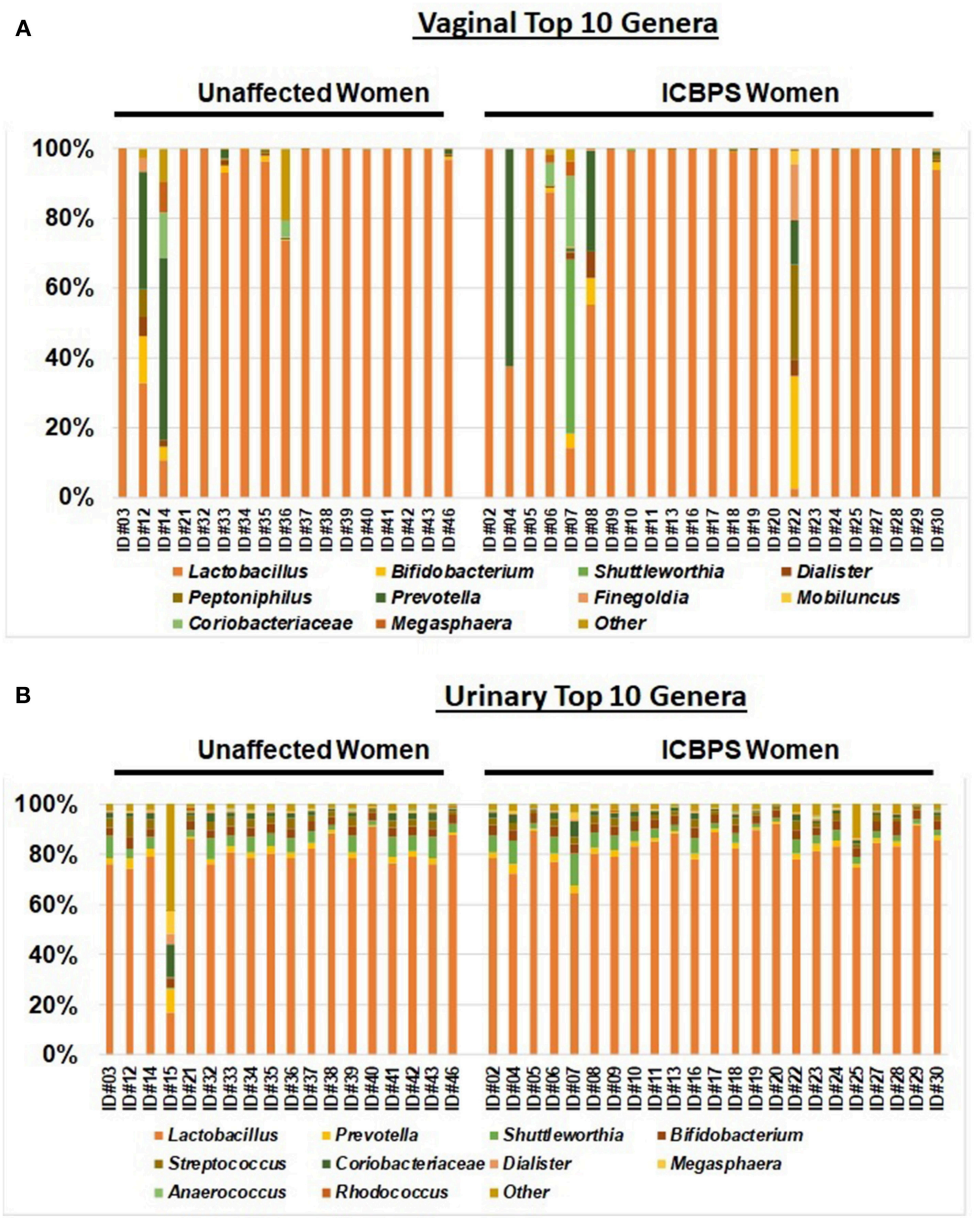
Stacked bar plots to the genus level of the top 10 genera for the vagina **(A)** and urinary samples **(B)** for the entire study population, with all genera not in the top 10 listed as “other.” Samples are sorted into unaffected (left) and ICBPS women (right) and labeled below with the individual woman's study identification number. Each color corresponds to a different genus, with *Lactobacilli* (orange) being predominant in most specimens.

Anaerobic predominance in the microbiome (the primary outcome) was rare, with only 12% (5/41; *n* = 2 ICBPS patients, *n* = 3 unaffected patients; *n* = 2 ICBPS) of women in the study having an anaerobic or fastidious genus as the primary contributor (>50% of reads) to either the vaginal or urinary microbiome. *Lactobacilli* was the dominant genus in the majority of urine (40/41, 98%) and vaginal (36/40, 85%) samples. The frequency of *Lactobacilli* dominance between urinary and vaginal samples trended toward the urine have more *Lactobacill*i dominance, albeit not statistically significant (*p* = 0.057). For vaginal samples, 2/23 ICBPS patients (9%) and 2/17 (12%) non-ICBPS patients (*p* = 0.99) had predominance of a non-*Lactobacilli* genus. For these 4 patients where *Lactobacilli* did not dominate the vaginal microbiome, dominant genera included *Prevotella* (*n* = 2 unaffected patients; *n* = 1 ICBPS) or *Shuttleworthia* (*n* = 1 ICBPS). For the urine, no ICBPS patients and only one non-ICBPS patient (1/18, 6%) had dominance of a non-*Lactobacilli* genus (44% *Prevotella*). Stacked bar plots of the vagina (Figure 4A) and the urinary samples (Figure 4B) are shown. At the OTU level, microbial species were also similar between groups for urine and vagina (Supplementary Table 1 and Supplementary Figure 1).

In comparing measures of alpha diversity between ICBPS and non-ICBPS groups on the logarithmic scale, no significant differences were seen in the urinary (*p* = 0.11) or vaginal Simpson indices (*p* = 0.70). For the Chao1 estimator of richness, the urine (*p* = 0.91) and the vagina (*p* = 0.64) were also similar. Shannon index and McIntosh evenness comparison between groups also showed no significant differences (Supplementary Table 2). ICBPS specimens demonstrated Kendall correlation between the vaginal and urinary Simpson indices (tau = 0.49, 95% CI 0.23 to 0.68, *p* < 0.01, but this was not seen for non-ICBPS women (tau = 0.27, 95% CI−0.03 to 0.58, *p* = 0.06). No other significant correlations were found between the urine and vagina for Chao1 or Simpson indices in either group.

Without FDR correction, we detected no significant relationships (Table 1) between the urinary Simpson index and the factors of smoking status (*p* = 0.11), OABq symptom index (*p* = 0.19) and quality of life (*p* = 0.20) scores, PISQ-IR condition impact score (*p* = 0.07), and mBIS score (*p* = 0.06). For vaginal samples, again without FDR correction, the PISQ-IR condition impact score (*p* = 0.15) and the mBIS score (*p* = 0.06) were non-significantly related to the Simpson index, and the CRADI-8 (*p* = 0.04) score was related to the vaginal Chao1 estimator (Table 1). However, after applying the FDR correction for marginal regression models at each site, none of these effects were significant at either the 0.05 or 0.20 FDR level.

## Discussion

In this cross-sectional pilot study of the urinary and vaginal microbiome comparing premenopausal women with ICBPS to those unaffected by ICBPS, we did not detect any significant differences in the vaginal or urinary microbiome. We did, however, note severe discrepancies in patient characteristics and pelvic and sexual function between the groups. These results indicate that ICBPS women have many risk factors for compromised pelvic health, and ICBPS is not a microbiome pathology.

Of note, the correlation between the diversity (Simpson index) of the vaginal and urinary samples was stronger in the ICBPS group than in unaffected women despite the use of clean-catch urinary specimens. ICBPS women are known to have altered epithelial function (Liu et al., 2012), so it is possible that this finding may indicate some difference in the natural barrier between the urinary and vaginal spaces in ICBPS women that this study was not designed to determine.

Unfortunately, this study lacks data indicating that alpha diversity correlates with validated urinary symptom scores, so ability to channel this finding at an intervention for ICBPS is limited. However, given that no prior study has explored the vaginal microenvironment in ICBPS, this data is novel and suggests a hypothesis that ICBPS allows more communication between the urinary and vaginal microbiomes.

This study demonstrates that the urinary and vaginal microbiomes in pre-menopausal women seen to have an overwhelming predominance of *Lactobacilli*, regardless of the presence of ICBPS. It is known that urine is not sterile and holds microbes that correspond greatly to the vaginal microbiome (Wolfe et al., 2012), but urine still holds less bacterial genomic material than the genitalia (Gottschick et al., 2017). *Lactobacilli* are associated with health of the vaginal microbiome (Stapleton, 2016; Lewis et al., 2017) and are predominant in the premenopausal urinary microbiome as well (Thomas-White et al., 2017). It appears that the vast majority of bacteria communicating between the urine from the vaginal environments are *Lactobacilli*, which is both statistically probable and physiologically sensible (the urethral epithelium has inherent defenses against more pathologic bacteria) (Stapleton, 2016). Nevertheless, the *Lactobacilli* composition in the urine or vagina was similar between ICBPS and non-ICBPS groups in this detailed study, so we cannot conclude that vaginal *Lactobacilli* disturbance is related to ICBPS pathology.

Strengths of the study include the use of detailed, reliable methods to determine the microbial composition of the genitourinary system. Genetic analyses are known to be more reliable than simple culture or enzyme-testing, particularly in urinary samples (Wolfe et al., 2012; Mouraviev and McDonald, 2018). This study also included the vaginal microbiomes in the analyses, as opposed to former studies that only investigate the urinary microbiome (Pearce et al., 2014; Thomas-White et al., 2017). As the urine and vaginal microbiomes are likely to be closely related, ignoring the vagina may fail to capture the entire pathophysiology. Finally, this study collected and investigated multiple possible confounders of the genitourinary microbiome, an intricate system that cannot be considered without patient factors.

Limitations of this study are mostly due to the smaller size of the study population, limiting power and ability to analyze subpopulations. The vaginal microbial environment in the premenopausal woman is extremely complex and changes with factors such as diet, the menstrual cycle, and vaginal medication use (Ravel et al., 2011; Nunn and Forney, 2016). We attempted to account for the days since a woman's LMP, but many women lacked these data, and the cycle day may be inaccurate or not correlate to the hormonal phase. Another limitation was our use of clean-catch urine. Past studies have found microbiome differences between clean-catch and catheterized urine specimens (Wolfe et al., 2012), but we cannot know what proportion of the urinary sample is vaginal contamination. Also, we did not explore viral or fungal data in these analyses, which focused only on the bacterial microbiome. Lastly, this population included a variety of genitourinary complaints within the sample group, making it difficult to isolate ICBPS pathology. Furthermore, the control population was mostly recruited from a subspecialty clinic where gynecological/urinary pathology is common regardless of the presence of ICBPS. ICBPS has varied presentation, and the “gold standard” used to validate some screening tools, the potassium challenge test, is known to be unreliable (Hanno et al., 2011). It is well known that ICBPS is a clinical diagnosis, but is extremely hard to diagnose with high specificity and sensitivity with current tools, given the large amoung of overlap with other pelvic pain syndromes and irritative voiding symptoms (Hanno et al., 2011; Patnaik et al., 2017). We gave preference to a questionnaire proven to correspond to disease severity and treatment response (Lubeck et al., 2001), which are most meaningful to patients and clinicians.

## Conclusion

The current study highlights that ICBPS is a disease with multiple layers of dysfunction. This pilot study did not detect alterations in vaginal or urinary microbiome associated with the presence of ICBPS. However, novel findings in this study could guide future investigation of altered urinary-vaginal microbial relationships in this disease state.

## Ethics Statement

University of Louisville Internal Review Board approved this study prior to any patient recruitment (IRB#13.0301), and all participating patients engaged in an informed consent conversation and signed a written consent document prior to participation or the collection of any patient data and samples.

## Author Contributions

KM and VJ contributed to the protocol and project development, data collection and management, data analysis, and the writing of the manuscript. ZL contributed to the protocol and project development, data collection and management, and the editing of the manuscript. RS contributed to the data collection and management, and data analysis. JG contributed to the data analysis and the editing of the manuscript. DH contributed to the data collection and management, and the editing of the manuscript.

### Conflict of Interest Statement

KM is a textbook editor for Elsevier publishing, and in the future will receive royalties for this textbook. The content of the textbook is unrelated to the content of this research article. The remaining authors declare that the research was conducted in the absence of any commercial or financial relationships that could be construed as a potential conflict of interest.

## Footnotes

^1^Bacterial Vaginosis. [cited 2018 April 4] Available online at: https://www.cdc.gov/std/tg2015/bv.htm.

^2^Castle T. JHeatChart. [cited 2018 Feb 23] Available online at: www.javaheatmap.com/.

## Supplementary Material

Supplementary Table 1 Operational taxonomic unit (OTU) level data showing the percentile of reads for each individual OUT for each sample, and the mean percentile for the sample group (ICBPS urine, unaffected urine, ICBPS vagina, unaffected vagina) for each OTU.

Supplementary Table 2 Table of alpha diversity measures (Chao1 richness measures, McIntosh evenness index, Simpson index, and Shannon index) at 5,000 sequences per sample for all specimens, with means displayed for each group (ICBPS and unaffected) for each group of samples (urinary and vaginal).

Supplementary Figure 1 OTU level data for ICBPS women for the urinary (ICBPS Image **A**; unaffected Image **B**) and vaginal spaces and unaffected women for the urinary and vagina (ICBPS Image **C**; unaffected Image **D**) spaces. Only the top 10 species for each group/space are shown. The y-axes are the percentiles of reads in the sample, and the x-axes display the individual patients and, on the far right, the mean percentile of reads in the sample for each species across the sample group. Note the large percentile of *Lactobacilli* species seen in the majority of samples.

Appendix 1 Details of study exclusion criteria.

## References

[B1] Abernethy M. G. Rosenfeld A. White J. R. Mueller M. G. Lewicky-Gaupp C. Kenton K. (2017). Urinary microbiome and cytokine levels in women with interstitial cystitis. Obstet. Gynecol. 129, 500–506. 10.1097/AOG.0000000000001892 28178051

[B2] Clemens J. Q. Elliott M. N. Suttorp M. Berry S. H. (2012). Temporal ordering of interstitial cystitis/bladder pain syndrome and non-bladder conditions. Urology 80, 1227–1231. 10.1016/j.urology.2012.06.059 23206765 PMC3804419

[B3] Clemens J. Q. Meenan R. T. O'Keeffe Rosetti M. C. Brown S. O. Gao S. Y. Calhoun E. A. (2005). Prevalence of interstitial cystitis symptoms in a managed care population. J. Urol. 174, 576–580. 10.1097/01.ju.0000165170.43617.be 16006901

[B4] Comeau A. M. Douglas G. M. Langille M. G. (2017). Microbiome helper: a custom and streamlined workflow for microbiome research. mSystems 2:e00127-16. 10.1128/mSystems.00127-16 28066818 PMC5209531

[B5] DeSantis T. Z. Hugenholtz P. Larsen N. Rojas M. Brodie E. L. Keller K.. (2006). Greengenes, a chimera-checked 16S rRNA gene database and workbench compatible with ARB. Appl. Environ. Microbiol. 72, 5069–5072. 10.1128/AEM.03006-05 16820507 PMC1489311

[B6] Edgar R. C. Haas B. J. Clemente J. C. Quince C. Knight R. (2011). UCHIME improves sensitivity and speed of chimera detection. Bioinformatics 27, 2194–2200. 10.1093/bioinformatics/btr381 21700674 PMC3150044

[B7] Gottschick C. Deng Z. L. Vital M. Masur C. Abels C. Pieper D. H.. (2017). The urinary microbiota of men and women and its changes in women during bacterial vaginosis and antibiotic treatment. Microbiome 5:99. 10.1186/s40168-017-0305-3 28807017 PMC5554977

[B8] Hanno P. M. Burks D. A. Clemens J. Q. Dmochowski R. R. Erickson D. Fitzgerald M. P.. (2011). AUA guideline for the diagnosis and treatment of interstitial cystitis/bladder pain syndrome. J. Urol. 185, 2162–2170. 10.1016/j.juro.2011.03.064 21497847 PMC9341322

[B9] Kanter G. Volpe K. A. Dunivan G. C. Cichowski S. B. Jeppson P. C. Rogers R. G.. (2017). Important role of physicians in addressing psychological aspects of interstitial cystitis/bladder pain syndrome (IC/BPS): a qualitative analysis. Int. Urogynecol. J. 28, 249–256. 10.1007/s00192-016-3109-2 27581769 PMC5292090

[B10] Karstens L. Asquith M. Davin S. Stauffer P. Fair D. Gregory W. T.. (2016). Does the urinary microbiome play a role in urgency urinary incontinence and its severity? Front. Cell. Infect. Microbiol. 6:78. 10.3389/fcimb.2016.00078 27512653 PMC4961701

[B11] Kopylova E. Noe L. Touzet H. (2012). SortMeRNA: fast and accurate filtering of ribosomal RNAs in metatranscriptomic data. Bioinformatics 28, 3211–3217. 10.1093/bioinformatics/bts611 23071270

[B12] Lewis F. M. Bernstein K. T. Aral S. O. (2017). Vaginal microbiome and its relationship to behavior, sexual health, and sexually transmitted diseases. Obstet. Gynecol. 129, 643–654. 10.1097/AOG.0000000000001932 28277350 PMC6743080

[B13] Liu H. T. Shie J. H. Chen S. H. Wang Y. S. Kuo H. C. (2012). Differences in mast cell infiltration, E-cadherin, and zonula occludens-1 expression between patients with overactive bladder and interstitial cystitis/bladder pain syndrome. Urology 80:225. 10.1016/j.urology.2012.01.047 22521193

[B14] Lozupone C. Knight R. (2005). UniFrac: a new phylogenetic method for comparing microbial communities. Appl. Environ. Microbiol. 71, 8228–8235. 10.1128/AEM.71.12.8228-8235.2005 16332807 PMC1317376

[B15] Lubeck D. P. Whitmore K. Sant G. R. Alvarez-Horine S. Lai C. (2001). Psychometric validation of the O'leary-Sant interstitial cystitis symptom index in a clinical trial of pentosan polysulfate sodium. Urology 57(6 Suppl. 1):62–66. 10.1016/S0090-4295(01)01126-8 11378052

[B16] Mercier C. B. A. Coissac E. (2013). SUMATRA and SUMACLUST: Fast and Exact Comparison and Clustering of Sequences. Available online at: http://metabarcoding.org/sumatra/ (accessed April 17, 2017).

[B17] Mouraviev V. McDonald M. (2018). An implementation of next generation sequencing for prevention and diagnosis of urinary tract infection in urology. Can. J. Urol. 25, 9349–9356. 29900824

[B18] Navas-Molina J. A. Peralta-Sanchez J. M. Gonzalez A. McMurdie P. J. Vazquez-Baeza Y. Xu Z.. (2013). Advancing our understanding of the human microbiome using QIIME. Meth. Enzymol. 531, 371–444. 10.1016/B978-0-12-407863-5.00019-8 24060131 PMC4517945

[B19] Nickel J. C. Stephens A. Landis J. R. Mullins C. van Bokhoven A. Lucia M. S.. (2016). Assessment of the lower urinary tract microbiota during symptom flare in women with urologic chronic pelvic pain syndrome: a MAPP network study. J. Urol. 195, 356–362. 10.1016/j.juro.2015.09.075 26410734 PMC4770794

[B20] Nunn K. L. Forney L. J. (2016). Unraveling the dynamics of the human vaginal microbiome. Yale J. Biol. Med. 89, 331–337. 27698617 PMC5045142

[B21] Patnaik S. S. Lagana A. S. Vitale S. G. Buttice S. Noventa M. Gizzo S.. (2017). Etiology, pathophysiology and biomarkers of interstitial cystitis/painful bladder syndrome. Arch. Gynecol. Obstet. 295, 1341–1359. 10.1007/s00404-017-4364-2 28391486

[B22] Pearce M. M. Hilt E. E. Rosenfeld A. B. Zilliox M. J. Thomas-White K. Fok C.. (2014). The female urinary microbiome: a comparison of women with and without urgency urinary incontinence. MBio 5, e01283–e01214. 10.1128/mBio.01283-14 25006228 PMC4161260

[B23] Ravel J. Gajer P. Abdo Z. Schneider G. M. Koenig S. S. McCulle S. L.. (2011). Vaginal microbiome of reproductive-age women. Proc. Natl. Acad. Sci. U S A. 108 (Suppl. 1), 4680–4687. 10.1073/pnas.1002611107 20534435 PMC3063603

[B24] Rognes T. Flouri T. Nichols B. Quince C. Mahe F. (2016). VSEARCH: a versatile open source tool for metagenomics. PeerJ 4:e2584. 10.7717/peerj.2584 27781170 PMC5075697

[B25] Siddiqui H. Lagesen K. Nederbragt A. J. Jeansson S. L. Jakobsen K. S. (2012). Alterations of microbiota in urine from women with interstitial cystitis. BMC Microbiol. 12:205. 10.1186/1471-2180-12-205 22974186 PMC3538702

[B26] Stapleton A. E. (2016). The vaginal microbiota and urinary tract infection. Microbiol. Spectr. 4:UTI-0025-2016. 10.1128/microbiolspec.UTI-0025-2016 28087949 PMC5746606

[B27] Thomas-White K. Forster S. C. Kumar N. Van Kuiken M. Putonti C. Stares M. D.. (2018). Culturing of female bladder bacteria reveals an interconnected urogenital microbiota. Nat. Commun. 9:1557. 10.1038/s41467-018-03968-5 29674608 PMC5908796

[B28] Thomas-White K. J. Kliethermes S. Rickey L. Lukacz E. S. Richter H. E. Moalli P.. (2017). Evaluation of the urinary microbiota of women with uncomplicated stress urinary incontinence. Am. J. Obstet. Gynecol. 216:55. 10.1016/j.ajog.2016.07.049 27498309 PMC5182144

[B29] Wolfe A. J. Toh E. Shibata N. Rong R. Kenton K. Fitzgerald M.. (2012). Evidence of uncultivated bacteria in the adult female bladder. J. Clin. Microbiol. 50, 1376–1383. 10.1128/JCM.05852-11 22278835 PMC3318548

[B30] Zhang J. Kobert K. Flouri T. Stamatakis A. (2014). PEAR: a fast and accurate Illumina Paired-End reAd mergeR. Bioinformatics 30, 614–620. 10.1093/bioinformatics/btt593 24142950 PMC3933873

