# Supplementary figures and images for: The Vaginal and Urinary Microbiomes in Premenopausal Women With Interstitial Cystitis/Bladder Pain Syndrome as Compared to Unaffected Controls: A Pilot Cross-Sectional Study
Source: Front Cell Infect Microbiol. 2019 Apr 8;9:92. doi: 10.3389/fcimb.2019.00092 (PMC6463740; doi:10.3389/fcimb.2019.00092)

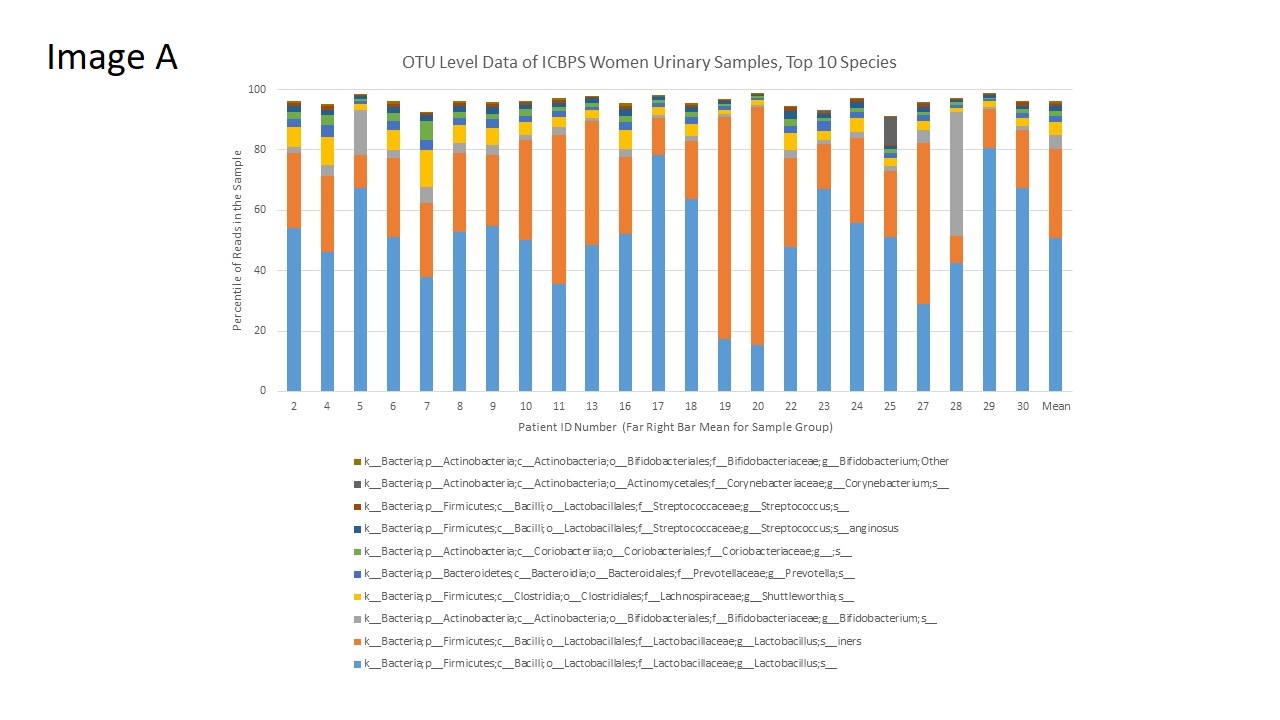

Supplement: Supplementary Figure 1 — OTU level data for ICBPS women for the urinary (ICBPS Image A; unaffected Image B) and vaginal spaces and unaffected women for the urinary and vagina (ICBPS Image C; unaffected Image D) spaces. Only the top 10 species for each group/space are shown. The y-axes are the percentiles of reads in the sample, and the x-axes display the individual patients and, on the far right, the mean percentile of reads in the sample for each species across the sample group. Note the large percentile of Lactobacilli species seen in the majority of samples. [file Image_1.jpg]

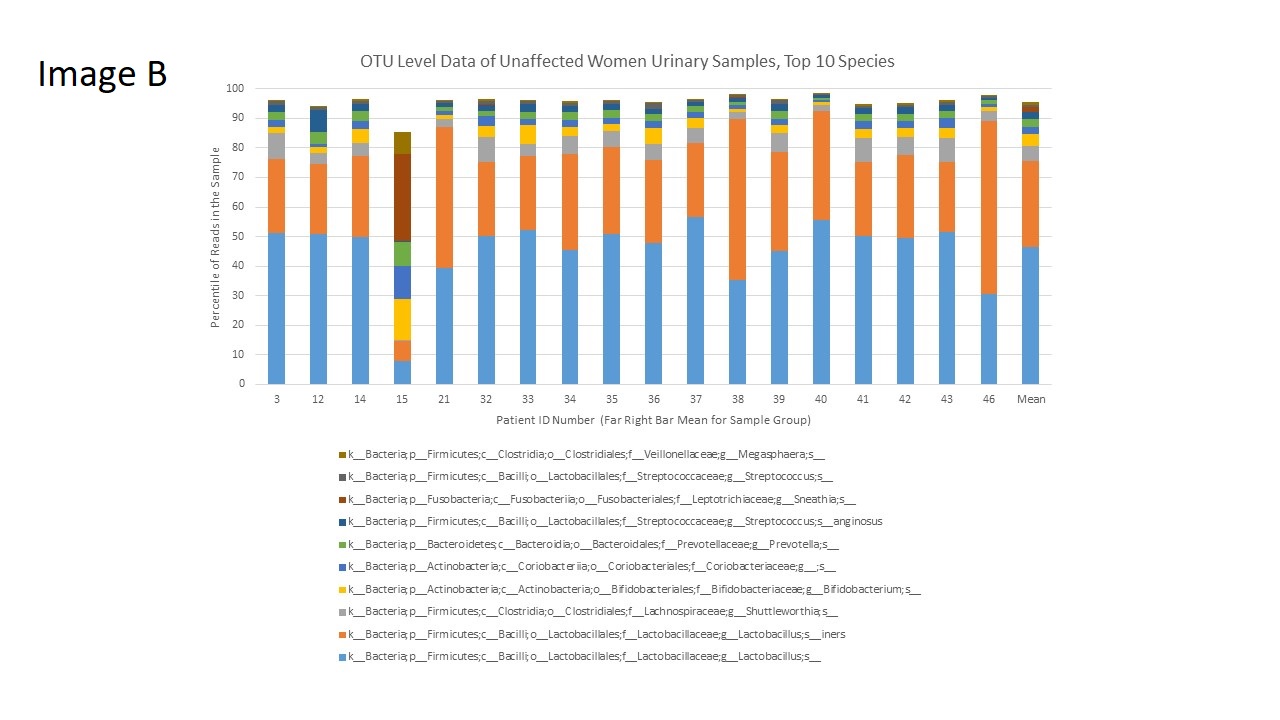

Supplement: Supplementary file 5 [file Image_2.jpg]

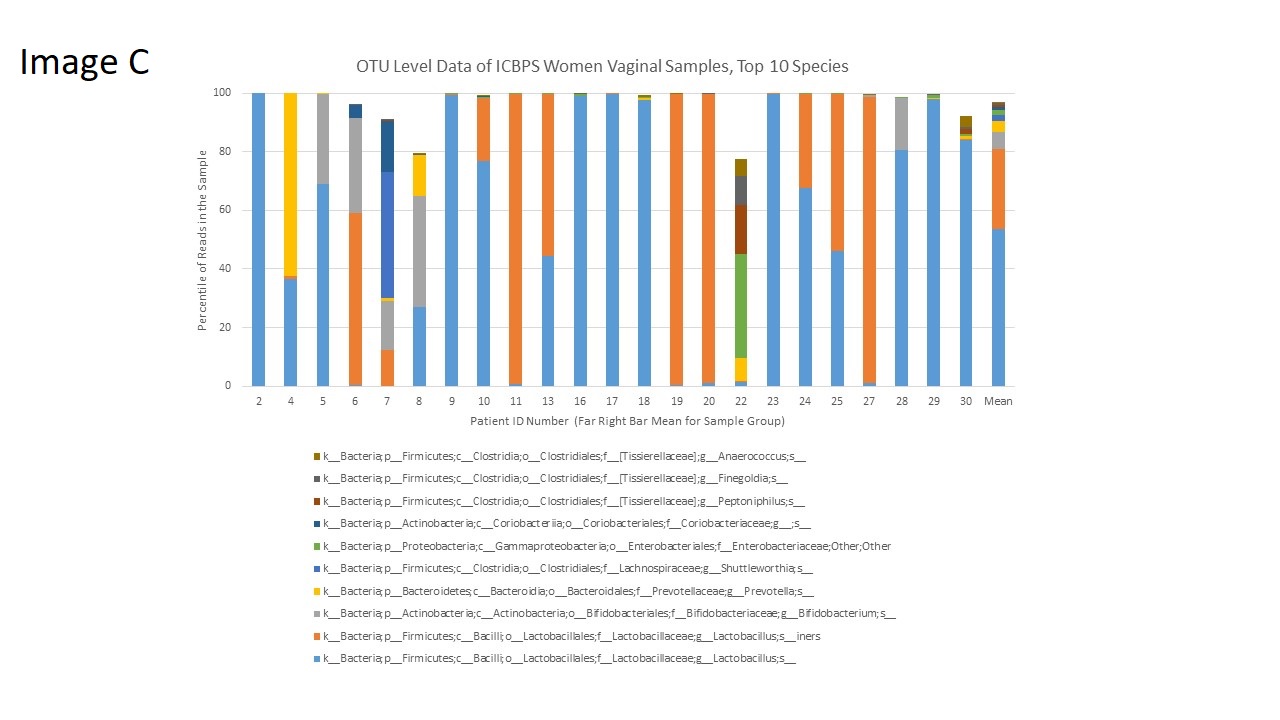

Supplement: Supplementary file 6 [file Image_3.jpg]

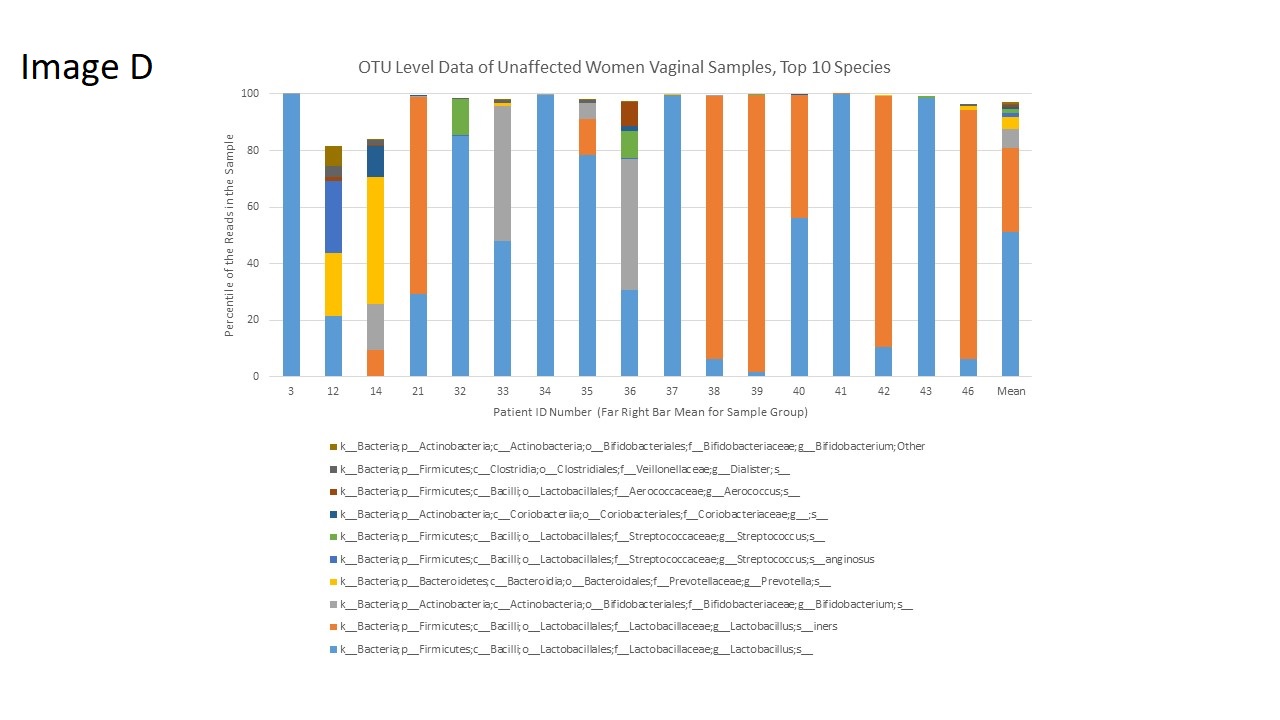

Supplement: Supplementary file 7 [file Image_4.jpg]
