# Supplementary material for: The Vaginal and Urinary Microbiomes in Premenopausal Women With Interstitial Cystitis/Bladder Pain Syndrome as Compared to Unaffected Controls: A Pilot Cross-Sectional Study
Source: Front Cell Infect Microbiol. 2019 Apr 8;9:92. doi: 10.3389/fcimb.2019.00092 (PMC6463740; doi:10.3389/fcimb.2019.00092)
Supplement: Appendix 1 — Details of study exclusion criteria. [file Data_Sheet_1.docx]

Appendix 1: Details of study exclusion criteria

| **Exclusion Criteria** | **Definition/Details** |
| --- | --- |
| Hormonally post-menopausal | Any one of the following:   - Bilateral past ovarian removal - ≥1 year since last menses if uterus in place - History of hysterectomy with ≥1 ovary in place and >1 year of menopausal symptoms - Laboratory confirmation of menopausal hormonal status (such as serum follicle stimulation hormone >25 IU/mL) |
| Current treatment for interstitial cystitis/bladder pain syndrome (ICBPS) applied directly to vagina or urine | Any one of the following in the last 2 weeks:   - Vaginal creams - Vaginal injections - Bladder instillations |
| Pelvic organ prolapse beyond the hymen | Measurement of >0 on any compartment of the pelvic organ prolapse quantification (POPQ) exam |
| Foreign bodies in the vagina or urinary system | Use or presence of the following in the last 2 weeks:   - Vaginal pessary - Vaginal hormone secreting device - Indwelling urinary catheter (transurethral or trans-abdominal) - Urethral stents   Vaginal surgery in the last 6 weeks or suture material in vagina at time of study visit |
| Evidence of active urinary tract infection | Positive microscopic urinalysis with at least one symptom:   - Positive nitrates, 2+ or more leukocyte esterase, large bacterial count, or combination   Positive urine culture with or without symptoms:   - ≥10^5 colony forming units (CFUs) on clean-catch specimen urine culture - ≥10^5 CFU’s of a single uropathogen on catheterized urine specimen |
| Recurrent or chronic bacterial vaginosis (BV) | >2 episodes within the last 12 months |
| Active known vaginal or cervical/pelvic infection | Any of the following in the last 2 weeks:   - Persistent symptoms of vaginal discharge, pain, itching, bleeding of new onset without workup or treatment - Known, laboratory proven vaginitis without adequate treatment - Treatment for known vaginitis or cervical/pelvic infection within the last 7 days or completed <7 days ago |
| Active vaginal ulcerative disease | Any of the following:   - Viral, traumatic, iatrogenic, or autoimmune ulcers of the vaginal walls or vulva at time of study visit - Active vaginal ulcerative disease (e.g. herpes simplex) with >2 episodes in the last year or last episode <1 month ago |
| Current daily antibiotic use | For reasons not listed above |
| Inability to understand or complete study materials | Inability to speak or read English or mental or cognitive disorder that prevents understanding of informed consent process |
